# Editorial: Emotions and sport performance

**DOI:** 10.3389/fpsyg.2023.1207623

**Published:** 2023-06-01

**Authors:** Lael Gershgoren, Jean-Charles Lebeau, Sicong Liu, Gershon Tenenbaum

**Affiliations:** ^1^The School of Behavioral Sciences, College of Management Academic Studies, Rishon LeTsiyon, Israel; ^2^School of Kinesiology, Ball State University, Muncie, IN, United States; ^3^Annenberg School of Communication, University of Pennsylvania, Philadelphia, PA, United States; ^4^Baruch Ivcher School of Psychology, Reichman University, Herzliya, Israel

**Keywords:** Individual Zone of Optimal functioning (IZOF), Individual Affect-related Performance Zones (IAPZ), emotional regulation, affect, mood

Competitive sports evoke a wide range of feelings and emotions. The study of emotions-performance linkage has been primarily guided by the Individual Affect-related Performance Zones (IAPZ; Kamata et al., 2002) and the Individual Zone of Optimal functioning (IZOF; Hanin, 2000) conceptual frameworks. The IAPZ operationalize the association between the intensity of positive and negative emotions and the athletic performance. Worth noting, the emotion-performance association highlighted in this approach is probabilistic, wherein performance level can be probabilistically modeled and predicted using the valence and intensity of emotions. Specifically, an individualized range of intensity (i.e., the zone) for certain emotions is more likely to be associated with one's optimal performance than intensity levels falling outside of such a range. Because several emotions can be experienced simultaneously, self-regulation is required from the athlete to remain in their emotion-related zones. Self-regulation of emotions depends on the performer's ability to facilitate, maintain or inhibit each emotion respectively. Ultimately, such an emotional regulation ability is key for optimizing precompetitive states and improve performance (Robazza et al., 2004).

Anxiety is the single most researched emotion in the sport psychology literature (e.g., Palazzolo, 2020; Kerr, 2021), perhaps at the expense of other emotions. Despite some efforts to address this gap (for a review, see Janelle et al., 2020), several other sport-related emotions, such as anger, shame, envy, pride, relief, and hope are yet to receive the attention they deserve. Two examples of additional common, yet under explored, emotions in sport are *frustration* (due to losing, underperforming, getting hurt, biased officiating, etc.; Tenenbaum et al., 1997) and *courage* (as coach Pep Guardiola said in the 2018 English cup-final half-time speech: “you have to learn to play football with courage”).

The six articles included in this special issue attempt to expand the knowledge on emotions and sport performance. Capturing emotions in sport requires the recognition of their triggers. Aligned with this aim, Kovács et al. qualitatively categorized the stressors that junior athletes, parents, and coaches feel in gymnastics. The emerging categories are discussed in light of Lazarus and Folkman's (1984) transactional model of stress and the mastery vs. performance -oriented goals. Remaining in youth sport, Morano et al. described the specialization stage of athletic development. In particular, the authors examined the interplay among self-perceptions, emotion-related experiences (e.g., feeling confident, focused, and determined), and burnout symptoms in adolescent athletes. Their findings can inform coaches and performance enhancement consultants in preventing burnout and preserving positive experiences that can contribute to long-term career success of junior athletes. Moving beyond youth sport settings, Parsons-Smith et al. utilized the mood profiles (i.e., iceberg, inverse iceberg, and Everest) to examine pre-race mood patterns in adult athletes categorized into age and gender cohorts. The six distinct mood profile clusters are discussed in depth in relation to performance and mental health. Next, Jacobs and Keegan explored the connection between emotional awareness and resilience in emergency services personnel, and outlined how their finding can benefit athletes performing under stressful conditions. From an applied perspective, Buchanan and Janelle investigated how slow, normal, and fast breathing frequencies affect motor performance under pleasant and unpleasant emotional conditions from an applied perspective. In light of the findings, the authors further discuss breathing regulation as an emotion regulation strategy. In this vein, Orbach and Blumenstein presented a mental training method aimed at achieving optimal emotional state. They described a theoretical and practical framework that fuses biofeedback training, periodization principle, and the Learning-Modification-Application model to refine the pre-competition, pre-performance, and post-performance routines in sports.

We believe that altogether the special issue extends the current state of the literature and promotes future research on emotions in sport. Some research directions include:

- the impact of various discrete emotions on athletic performance,
- emotions evoked when athletes are injured and during their rehabilitation process,
- emotions in Esports,
- team emotions (e.g., interpersonal emotional influences and emotional contagion),
- emotional intelligence effects on athletes' wellbeing,
- emotional intelligence among coaches,
- post-performance emotions,
- innovational intervention programs for emotional regulation,
- mood, affect, and their relations to sport performance.

These research endeavors, so we believe, offer new directions in studying individual and team-shared emotions and their behavioral and performance consequences in sport and other domains.

## Author contributions

All authors listed have made a substantial, direct, and intellectual contribution to the work and approved it for publication.
